# Radial-Side First Technique: Revisiting the Radial Forearm Flap Dissection in a Cadaveric Model

**DOI:** 10.7759/cureus.105296

**Published:** 2026-03-16

**Authors:** Anas Elsayed, Pedro Fuenmayor, Ricardo Castrellon

**Affiliations:** 1 Plastic Surgery, Philadelphia Collage of Osteopathic Medicine, Philadelphia, USA; 2 Plastic and Reconstructive Surgery, Larkin Community Hospital, South Miami, USA

**Keywords:** cadaver dissection, operative technique, pedicled fasciocutaneous flap, radial artery, radial forearm flap, radial-side first dissection

## Abstract

This study details an anatomical dissection of the radial forearm flap, a cornerstone in reconstructive surgery. It explores an alternative approach by initiating dissection from the radial side, contrary to the traditional ulnar-side start. This method demonstrates the feasibility, safety, and efficacy of achieving early access to key structures, including the radial artery, venae comitantes, nerves, veins, and tendons. A dissection was performed on a formalin-fixed human specimen. The flap was designed around the radial artery. Radial-side dissection provided immediate access to the distal radial artery and facilitated preservation of the superficial radial nerve. Subsequent ulnar-side dissection proceeded suprafascially from proximal to distal, identifying the palmaris longus and flexor carpi radialis. Critical structures, including the lateral antebrachial cutaneous nerve, were meticulously isolated and preserved. Standard surgical instruments were used for dissection and ligation. As a cadaveric study, direct applicability to live surgery may be limited due to variances in patient anatomy, tissue viability, and intraoperative dynamics. Surgeon-specific factors such as experience and preference were not assessed. Initiating dissection from the radial side facilitates early control of critical vasculature and enables precise preservation of neural and vascular elements. This approach offers significant procedural benefits; however, further clinical studies are necessary to validate its efficacy across the variability inherent in live surgical procedures.

## Introduction

The radial forearm fasciocutaneous flap, based on the radial artery and its venae comitantes, provides reliable, thin, and pliable tissue for reconstructing complex defects of the elbow, wrist, and hand [1]. Its versatility allows for antegrade or reverse flow designs, making it a workhorse for upper-extremity soft tissue coverage, with primary healing achieved in approximately 95% of cases [1,2].

Despite its reliability, flap harvest carries potential morbidity. Donor-site complications, including tendon exposure (13%) and sensory dysfunction from injury to the superficial branch of the radial nerve (SBRN) (32%), remain significant concerns [3,4]. Conventional harvest typically begins on the ulnar side, which may delay direct visualization of the radial artery pedicle and defer SBRN identification, potentially placing these structures at risk [5].

In an effort to optimize safety, we demonstrate a radial-side first approach, prioritizing early vascular pedicle control and SBRN preservation using consistent anatomical landmarks. This cadaveric study describes the technique stepwise, highlighting its anatomic rationale and potential clinical advantages for both trainees and experienced surgeons.

## Technical report

Materials and methods

One formalin-fixed cadaveric upper extremity was used. The flap was designed over the distal two-thirds of the forearm, centered on the radial artery. A curvilinear incision was marked, extending proximally and stopping approximately 1 cm short of the antecubital fossa to facilitate proximal pedicle dissection. The dissection was performed following a stepwise technique, comprehensively detailed in Video 1.

**Video 1:** Radial-Side First Radial Forearm Flap Dissection

Radial Incision and Early Pedicle Control 

Skin incision was made along the pre-marked radial border. Subcutaneous dissection immediately identified the cephalic vein, which was preserved. The distal radial margin of the flap was elevated, allowing prompt identification of the SBRN coursing superficial to the brachioradialis tendon. This was dissected and preserved. The brachioradialis tendon was retracted radially, revealing the lateral intermuscular septum. Blunt dissection through the septum provided immediate and direct access to the radial artery and paired venae comitantes, achieving early vascular control.

Ulnar Dissection

The ulnar incision was completed, and the flap was elevated in a suprafascial plane. The flexor carpi ulnaris (FCU) and palmaris longus (PL) tendons were visualized, and their paratenon was preserved. The dissection continued until reaching the vascular pedicle.

Vascular Pedicle Elevation

The plane of dissection was connected from radial to ulnar, deep to the antebrachial fascia to protect the pedicle. After distal ligation of the vascular structures, the pedicle was elevated proximally. Perforating vessels were carefully divided when necessary. 

Identification of Additional Structures

The lateral antebrachial cutaneous nerve (LACN), a terminal branch of the musculocutaneous nerve, was identified proximal to the radial artery and preserved. The cephalic vein was dissected free. The flap was thus completely elevated on its pedicle.

The entire procedure was recorded using high-definition overhead and surgical-point-of-view cameras. Narration describes key technical points and anatomical relationships.

Results

The radial-side first approach provides a logical and efficient dissection. The most significant advantage was the prompt identification and control of the vascular pedicle, securing the flap's viability from the beginning. The SBRN is visualized and preserved before extensive dissection, minimizing the risk of iatrogenic transection or traction injury. The LACN was easily identified and protected later in the procedure.

Following initial pedicle control, ulnar-side dissection is significantly simplified. Suprafascial flap elevation progresses efficiently, providing excellent exposure of the FCU and PL tendons while preserving paratenon, to minimize donor site morbidity [6,7]. The antebrachial fascia remains largely intact, with disruption limited to the vascular pedicle for flap elevation. The cephalic vein is readily identified and may be harvested to augment venous outflow. Overall, this technique provides a reproducible sequence that minimizes blind dissection and secures early control of the pedicle and important structures.

## Discussion

The radial forearm flap (RFF) remains a cornerstone for upper-extremity reconstruction [1,2]. However, its harvest can be challenging. The traditional ulnar-side first approach, while effective, has inherent limitations. By beginning dissection away from the pedicle, the surgeon may encounter variable perforators before identifying the flap's axial blood supply, potentially increasing the risk of inadvertent injury during the early phases [5]. The radial-side first technique inverts this paradigm, representing a core principle of anatomic surgery: identify and control the most critical structures first.

This approach offers several compelling advantages that align with the goals of modern reconstructive surgery: efficiency, safety, and minimized morbidity. Early control of the radial artery establishes a clear and secure reference point for the procedure, allowing subsequent ulnar-side dissection toward a known visualized structure. This strategy minimizes pedicle injury risk and early flap loss while improving efficiency in free and pedicled reconstructions.

Latrogenic SBRN injury is a recognized source of donor-site morbidity, causing painful neuromas or dorsal-hand sensory deficits [3,8]. Radial-side first shifts SBRN preservation from reactive to proactive. Early vascular identification also serves an educational purpose, guiding trainees through a reproducible, stepwise harvest sequence and standardizing critical steps [9].

Additionally, the subsequent ulnar elevation in a suprafascial plane is more intuitively executed. The suprafascial technique preserves fascia and subcutaneous tissue, which is crucial for improved skin graft take, reduced healing time, superior cosmetic outcomes, and for reducing donor-site morbidity [6,7,10]. 

In modern reconstructive surgery, minimizing flap failure, reducing donor-site morbidity, and simplifying flap elevation are key goals. By addressing traditional pitfalls, the radial-side first technique enhances both safety and efficiency, reinforcing its value and supporting its continued use [11,12].

Limitations

The principal limitation of this study is its basis in cadaveric dissection, which cannot replicate live tissue perfusion, bleeding, or elasticity. Consequently, safety advantages can only be hypothesized. Validation requires clinical studies in living patients, where outcomes such as flap perfusion, operative time, donor-site morbidity, and SBRN injury rates can be systematically evaluated. 

## Conclusions

The radial-side first technique offers a rational, refined approach to RFF harvest. Prioritizing early vascular control and SBRN preservation addresses key limitations of the traditional ulnar-side first method. It enhances safety, efficiency, and educational value as it serves as an excellent framework. We advocate for its consideration in clinical practice and believe it should be integrated into the surgical repertoire as a standard technique for flap elevation.
